# No difference in cerebral perfusion between the wild-type and the 5XFAD mouse model of Alzheimer’s disease

**DOI:** 10.1038/s41598-022-26713-x

**Published:** 2022-12-22

**Authors:** Drew R. DeBay, Tân-Trào Phi, Chris V. Bowen, Steven C. Burrell, Sultan Darvesh

**Affiliations:** 1 Department of Medical Neuroscience, Dalhousie University, Halifax, NS Canada; 2 Department of Diagnostic Radiology, Dalhousie University, Halifax, NS Canada; 3 Biomedical Translational Imaging Centre (BIOTIC), IWK Health Centre, Halifax, NS Canada; 4 Department of Medicine (Neurology and Geriatric Medicine), Halifax, NS Canada; 5 Department of Chemistry and Physics, Mount St. Vincent University, Halifax, NS Canada

**Keywords:** Alzheimer's disease, Molecular imaging, Animal disease models, Alzheimer's disease, Brain

## Abstract

Neuroimaging with [2,2-dimethyl-3-[(2*R*,3*E*)-3-oxidoiminobutan-2-yl]azanidylpropyl]-[(2*R*,3*E*)-3-hydroxyiminobutan-2-yl]azanide;oxo(^99^Tc)technetium-99(3+) ([^99m^Tc]HMPAO) single photon emission computed tomography (SPECT) is used in Alzheimer’s disease (AD) to evaluate regional cerebral blood flow (rCBF). Hypoperfusion in select temporoparietal regions has been observed in human AD. However, it is unknown whether AD hypoperfusion signatures are also present in the 5XFAD mouse model. The current study was undertaken to compare baseline brain perfusion between 5XFAD and wild-type (WT) mice using [99mTc]HMPAO SPECT and determine whether hypoperfusion is recapitulated in 5XFAD mice. 5XFAD and WT mice underwent a 45 min SPECT scan, 20 min after [^99m^Tc]HMPAO administration. Whole brain and regional standardized uptake values (SUV) and regional relative standardized uptake values (SUVR) with whole brain reference were compared between groups. Brain perfusion was similar between WT and 5XFAD brains. Whole brain [^99m^Tc]HMPAO retention revealed no significant difference in SUV (5XFAD, 0.372 ± 0.762; WT, 0.640 ± 0.955; p = 0.536). Similarly, regional analysis revealed no significant differences in [^99m^Tc]HMPAO metrics between groups (SUV: 0.357 ≤ p ≤ 0.640; SUVR: 0.595 ≤ p ≤ 0.936). These results suggest apparent discrepancies in rCBF between human AD and the 5XFAD model. Establishing baseline perfusion patterns in 5XFAD mice is essential to inform pre-clinical diagnostic and therapeutic drug discovery programs.

## Introduction

Regional activation of neural elements (neurons and glia) in the brain evokes a closely regulated hemodynamic response, supplying blood that contains energy substrates, oxygen, and glucose, to satisfy local metabolic requirements^1,2^. This proportional enhancement of regional cerebral blood flow (rCBF) in activated areas of the brain reflects the neurovascular coupling phenomenon, highlighting the close link between neural activity, rCBF, and metabolism^3^. The exact mechanisms governing cerebral hemodynamics are complex and, in general, are poorly understood. Various physiological parameters influence cerebral blood flow which include mean arterial blood pressure, intracranial blood pressure, cerebral perfusion pressure (CPP), cerebrovascular resistance, and venous outflow among others^2^. Cerebral autoregulation orchestrates the interplay of various mediators at the molecular level to control perfusion homeostasis in the brain. In periods of neuronal activity, increased oxidative metabolism by neurons results in the extracellular excretion of ions and metabolites such as hydrogen ions (H^+^), CO_2_, and adenosine which contribute to vasodilation and the observed increase in rCBF^1^.

This neurovascular coupling phenomenon forms the basis of various neuroimaging techniques that serve as surrogate measures of brain function. This includes radiotracers for brain perfusion and metabolism that have been devised including perfusion-based Single Photon Emission Computed Tomography (SPECT) using [2,2-dimethyl-3-[(2*R*,3*E*)-3-oxidoiminobutan-2-yl]azanidylpropyl]-[(2*R*,3*E*)-3-hydroxyiminobutan-2-yl]azanide;oxo(^99m^Tc)technetium-99(3+) ([^99m^Tc]HMPAO) and metabolism-based Positron Emission Tomography (PET) using 2-deoxy-2[^18^F]fluoro-D-glucose ([^18^F]FDG).

Several radiotracers have been developed to evaluate rCBF in the brain. These have included SPECT imaging with ^133^Xenon (Xe) gas and ^123^I *p*-iodo-*N*-isopropylamphetamine (^123^IMP) and PET imaging with ^15^Oxygen, among others^1^. Various pitfalls have hampered the widespread clinical utility of such agents, including rapid clearance or redistribution within the brain, poor Signal-to-Noise Ratio (SNR) and low-resolution images. ^99m^Tc-based SPECT agents have become the mainstay to evaluate brain perfusion, including [^99m^Tc]HMPAO^4,5^ and (2*R*)-3-ethoxy-2-[2-[(2*R*)-1-ethoxy-1-oxo-3-sulfidopropan-2-yl]azanidylethylamino]-3-oxopropane-1-thiolate;oxo(^99^Tc)technetium-99(3+) ([^99m^Tc]ECD)^1^. [^99m^Tc]HMPAO and [^99m^Tc]ECD are small (< 500 Daltons) lipophilic molecules that readily cross the blood–brain barrier (BBB) and are retained in cells of the brain upon conversion into hydrophilic compounds^6^. Both radiotracers possess similar pharmacokinetic characteristics, reaching peak activity in the brain at approximately 2 min post-injection ([^99m^Tc]HMPAO, 2–3%ID; [^99m^Tc]ECD, 4–7%ID) and maintain a fixed flow-dependent distribution pattern in the brain (over several hours) that is proportional to rCBF at the time of injection^4^. Significant prolonged brain retention is seen with both tracers ([^99m^Tc]HMPAO, 85–88% by 15 min, 73% by 24 h; [^99m^Tc]ECD, 86–88% by 1 h and subsequent 6%/hour washout), with rapid washout of background tissues providing high gray-to-white matter contrast ([^99m^Tc]HMPAO, 2–3:1; [^99m^Tc]ECD, 4:1) and an imaging window of at least 2 h post injection ([^99m^Tc]HMPAO, up to 4 h; [^99m^Tc]ECD, up to 2 h).

Brain retention of [^99m^Tc]HMPAO has been thought to largely reflect an intracellular interaction with glutathione, an antioxidant which comprises most of all free thiols in mammalian tissue^6,7^. ^99m^Tc forms a chelated lipophilic complex with HMPAO and, when injected intravenously, readily crosses the BBB and subsequently is trapped intracellularly upon a glutathione-dependent conversion of the primary [^99m^Tc]HMPAO complex to a non-diffusible hydrophilic form^5,6,8^.

In neurodegenerative diseases such as Alzheimer’s disease (AD), perfusion neuroimaging with [^99m^Tc]HMPAO SPECT has been used as an ancillary test in AD diagnosis to evaluate regional cerebral perfusion^9^. As cerebral blood flow is closely coupled to neuronal activity, the activity distribution of [^99m^Tc]HMPAO has been used as a surrogate marker of neuronal activity levels in certain brain areas where specific hypoperfusion patterns have been observed in AD. For example, these hypoperfusion patterns have been observed in the posterior cingulate, precuneus, and temporoparietal cortex^10^. In general, perfusion SPECT has a moderate diagnostic value in AD (sensitivity/specificity = 80%/85%)^9^.

Few studies of SPECT perfusion have been carried out in mouse models^11^. For example, SPECT perfusion imaging of [^99m^Tc]HMPAO in a mouse model provided sufficient sensitivity to detect age-related differences in rCBF^11^.

It is not clear whether the patterns of human temporoparietal hypometabolism are recapitulated in animal models of AD that are commonly used for diagnostic and therapeutic drug development. The 5XFAD mouse is an AD transgenic mouse model of amyloidosis based on familial AD (FAD) mutations^12^. This model overexpresses mutant human amyloid precursor protein (*APP*), *APP*_*695*_, with the Swedish (K670N, M671L), Florida (I716V), and London (V717I) FAD mutations, as well as human presenilin 1 (PS1) with two FAD mutations (M146L and L286V). This mouse model of brain amyloidosis has a very aggressive course, exhibiting Aβ deposition accompanied by astrocytosis and microgliosis as early as 2 months of age with commensurate increases over disease progression^12^. In addition, a number of similarities between this AD animal model and human AD have been documented, including loss of synaptic markers observed at 9 months, cognitive impairment as early as 4 months^12,13^, and association of the AD-specific enzyme butyrylcholinesterase (BChE) with β-amyloid pathology^14,15^.

The aim of the current study was to evaluate the performance of [^99m^Tc]HMPAO in distinguishing perfusion patterns in the 5XFAD mouse model compared to the corresponding wild-type (WT) strain with the view to investigate whether these animal models recapitulate what is observed in the human AD condition.

## Results

**Figure 1:**
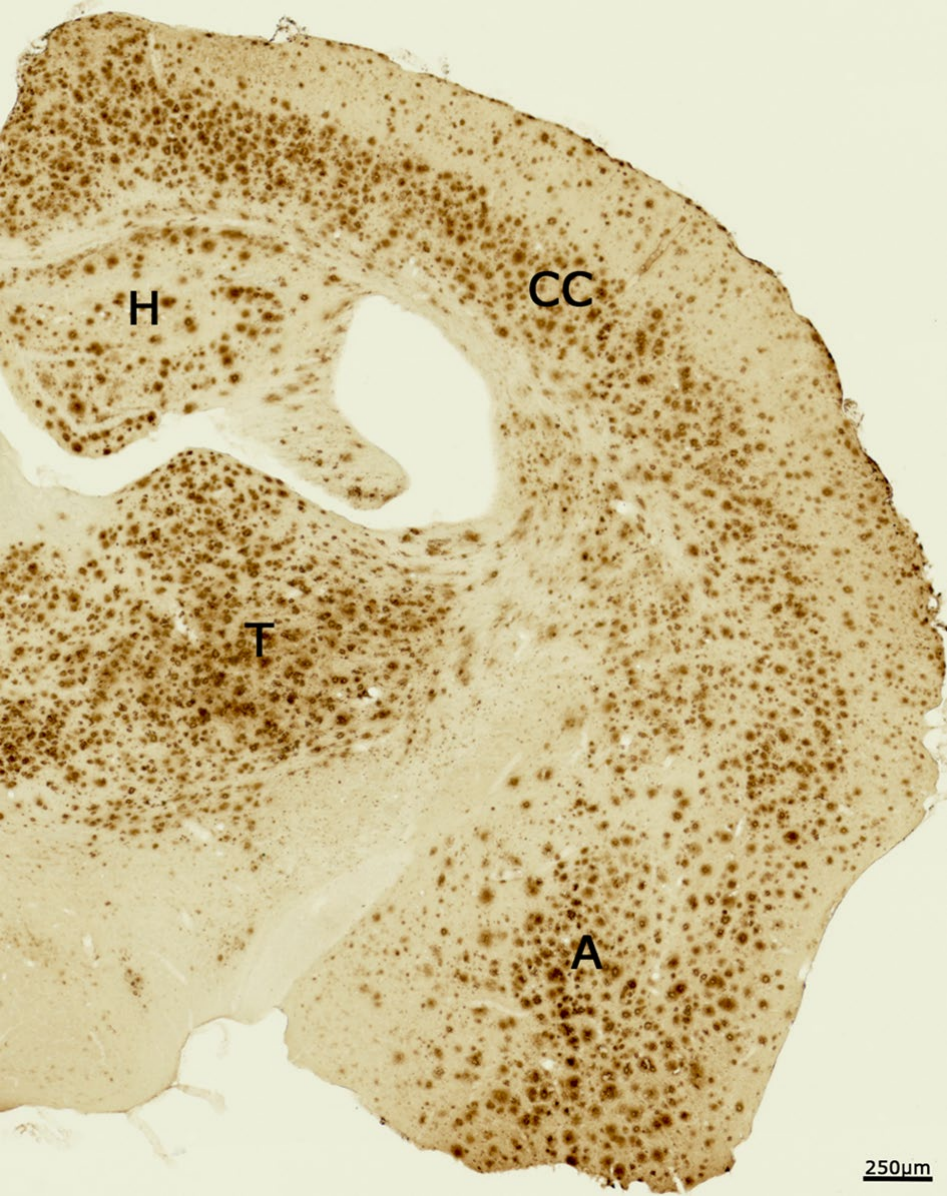
Photomicrograph showing immunohistochemical staining β-amyloid in the brain of the 5XFAD mouse used in this study. There is significant accumulation of β-amyloid plaques in a number of brain regions including the cerebral cortex (CC), hippocampus (H), thalamus (T), and amygdala (A). Scale bar 250 μm.

By 11 months of age in the 5XFAD mouse, there is significant accumulation of β-amyloid plaques (Fig. 1). The accumulation is robust in a number of regions including the cerebral cortex, thalamus, hippocampal formation, and amygdala. Following administration and 20 min of uptake of [^99m^Tc]HMPAO, a 40 min static SPECT scan was performed followed by computed tomography (CT) to evaluate regional brain perfusion in 5XFAD and WT mice, facilitated by magnetic resonance imaging (MRI) co-registration to a digital mouse atlas^16^.

### [^99m^Tc]HMPAO perfusion SPECT

**Figure 2:**
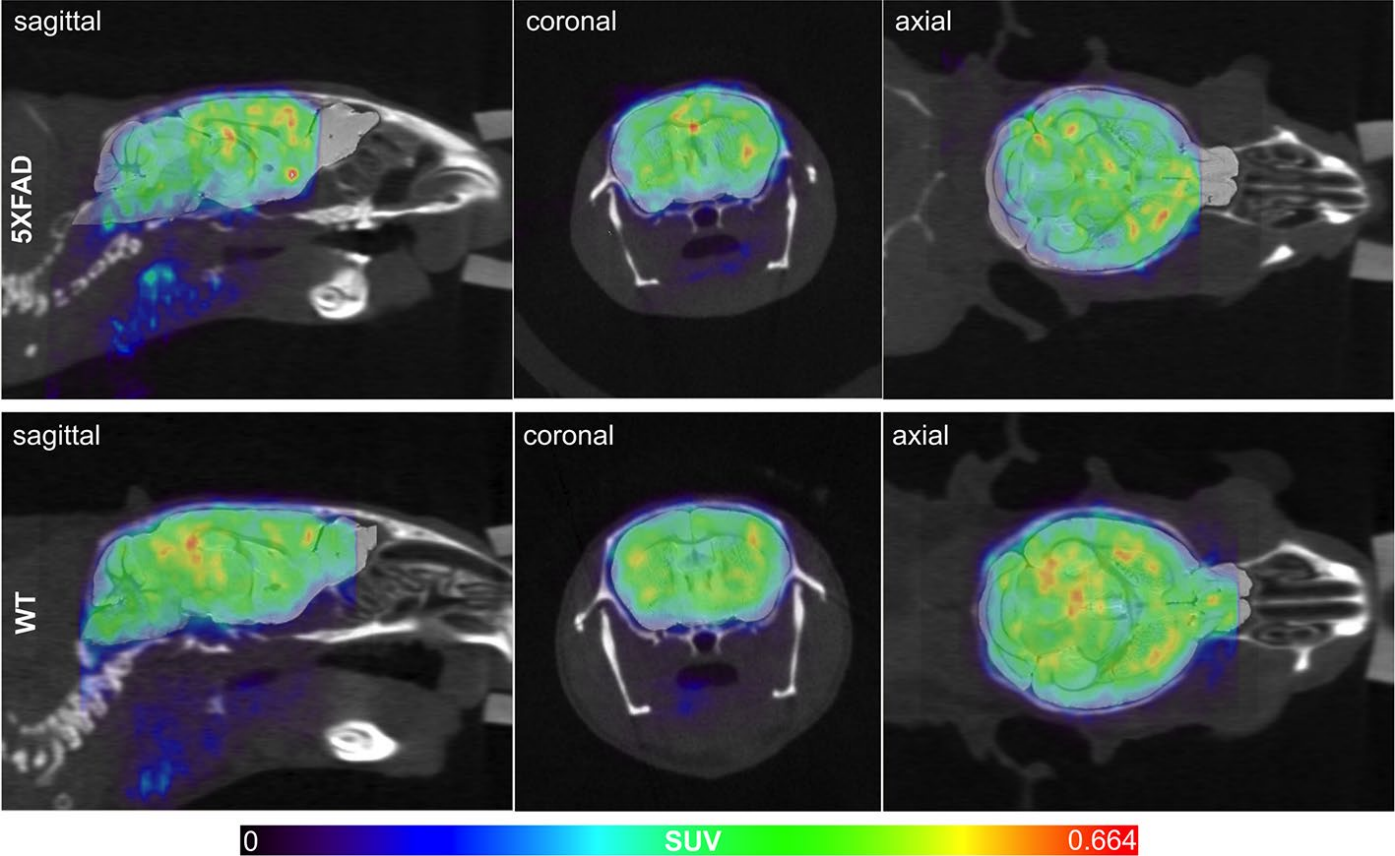
[^99m^Tc]HMPAO Single Photon Emission Computed Tomography/Computed Tomography (SPECT/CT) images are co-registered with a mouse-specific (warped) standard MR brain in sagittal, coronal, and axial planes for representative 5XFAD (top) and WT (bottom) mice. A similar distribution of [^99m^Tc]HMPAO radiotracer was observed between 5XFAD and WT mice. SPECT images were set to a common scale of 0–0.663 standardized uptake value (SUV) units.

[^99m^Tc]HMPAO SPECT scans generated sufficient image quality in both 5XFAD and WT mice, permitting appropriate image analyses and evaluation. SPECT scans showed similar general patterns of uptake on visual inspection (Fig. 2). Whole brain and regional evaluation of [^99m^Tc]HMPAO perfusion was carried out using standardized uptake value (SUV) and relative standardized uptake value (SUVR) metrics, the results of which are described below.

### Whole brain perfusion

**Figure 3:**
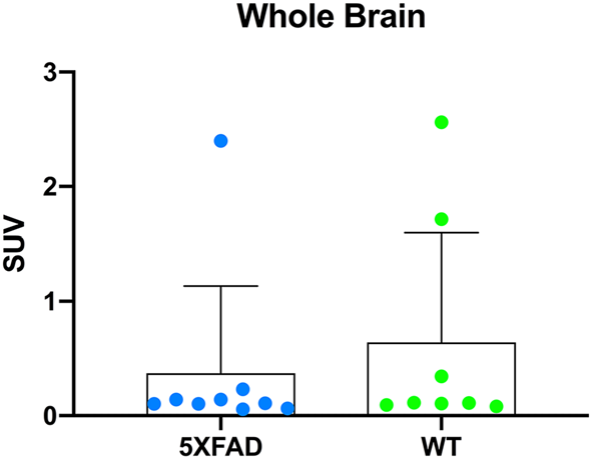
Whole brain [^99m^Tc]HMPAO SPECT perfusion measured via a standardized uptake value (SUV) in 5XFAD (blue) and WT (green) mice at 11.5 ± 0.2 months of age. No significant differences in whole brain perfusion were observed between 5XFAD (0.372 ± 0.762) and WT (0.640 ± 0.955) mice (p = 0.536). Individual subjects are represented by colored circles and the bar plot indicates Mean ± SD.

Whole brain uptake of [^99m^Tc]HMPAO revealed no significant differences in brain perfusion SUVs between 5XFAD (0.372 ± 0.762) and WT (0.640 ± 0.955) mice (p = 0.536) (Fig. 3). Three outliers were consistently observed in whole brain and regional SUV metrics (1 5XFAD and 2 WT), which were 2 standard deviations above the mean. Separate analysis (data not shown) comparing 5XFAD and WT with outliers removed was investigated, yielding the same conclusions, thus all data was included for the current analysis.

### Regional perfusion

**Figure 4:**
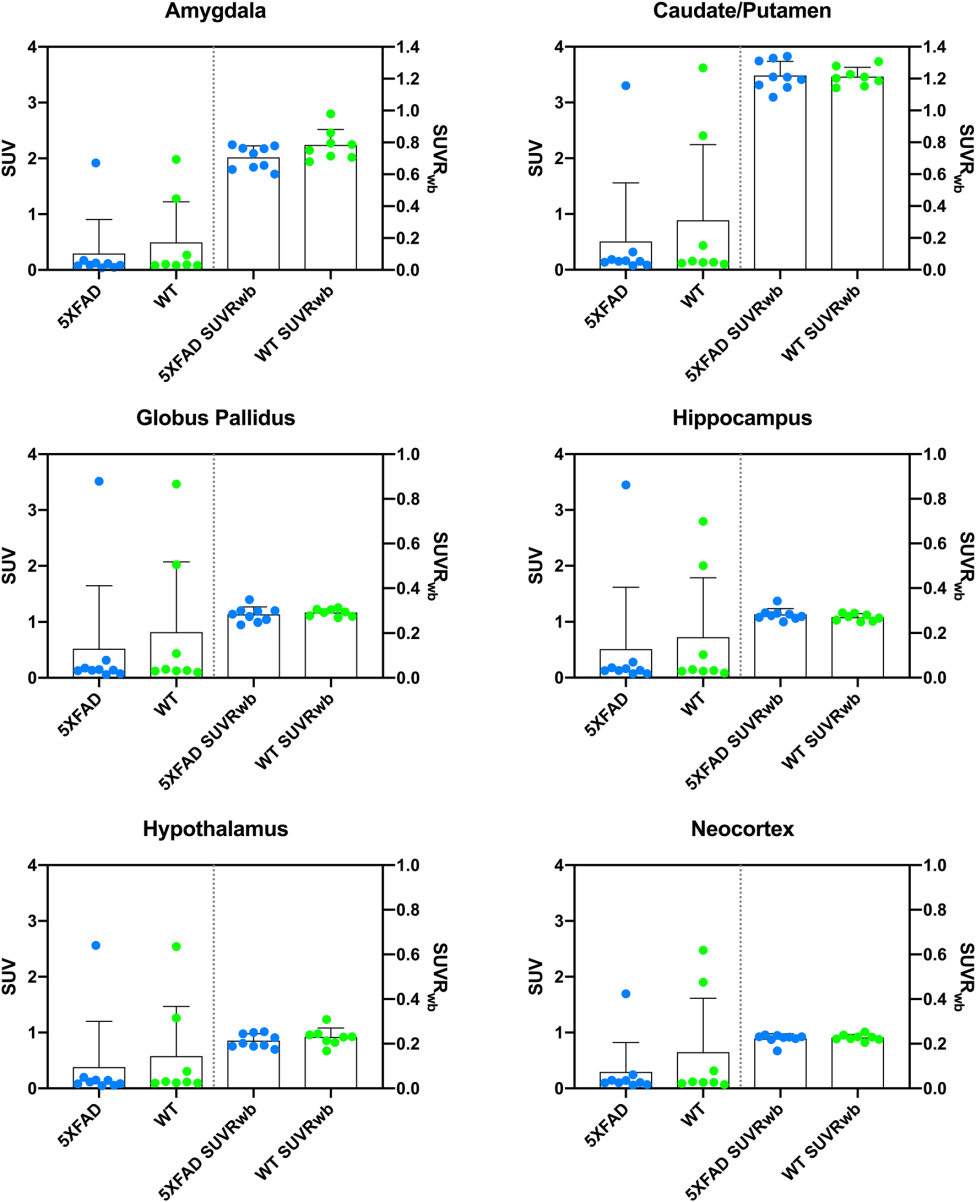
Regional [^99m^Tc]HMPAO SPECT perfusion measured in 5XFAD (blue) and WT (green) mice at 11.2 months of age. Standardized uptake values (SUV; left) and corresponding relative standardized uptake values with whole brain reference region (SUVR_wb_; right) were evaluated in the amygdala, caudate/putamen, globus pallidus, hippocampus, hypothalamus, and neocortex. No significant differences in regional perfusion were observed between 5XFAD and WT mice in any of the regions for either SUV or SUVR metrics (0.357 ≤ p ≤ 0.936). Individual subjects are represented by colored circles and the bar plot indicates mean ± SD.

A number of relevant brain regions-of-interest (ROIs) were investigated including amygdala, caudate/putamen, globus pallidus, hippocampus, hypothalamus, and neocortex. Among the regions evaluated, SUV values ranged from 0.296 ± 0.610 to 0.520 ± 1.125 for 5XFAD and from 0.497 ± 0.727 to 0.818 ± 1.253 for WT mice (Fig. 3). For SUVR metrics of the same regions, the values ranged from 0.706 ± 0.024 to 1.221 ± 0.089 for 5XFAD and from 0.784 ± 0.098 to 1.213 ± 0.057 for WT mice (Fig. 4). Regardless of the [^99m^Tc]HMPAO perfusion metric (SUV vs SUVR), no significant differences among brain regions were observed between 5XFAD and WT mice (SUV: 0.357 ≤ p ≤ 0.640; SUVR: 0.595 ≤ p ≤ 0.936) (Fig. 4).

## Discussion

The objective of the current study was to investigate the utility of a brain perfusion imaging agent [^99m^Tc]HMPAO in the 5XFAD mouse model. Given the widespread use of the 5XFAD mouse model in development of AD diagnostic and therapeutic agents, characterizing its brain function is essential to further our understanding of the effects of disease progression on important physiological parameters including cerebral blood flow. Cerebral blood flow is a particularly relevant parameter in radiotracer development as perfusion is directly related to tracer transfer from arterial blood to the brain.

Herein, no differences in baseline brain perfusion were observed between 5XFAD and WT mice at 11 months of age at the whole brain level nor in subsequent regional analyses using SUV and SUVR metrics. Subtle differences in animal-to-animal and day-to-day variations in physiology could significantly impact the uptake of the tracer into various organs, including the brain. Though anesthetic and temperature were controlled for, and respiratory rates were measured consistently throughout the course of study, heart rate and other physiological parameters were not evaluated, which could introduce a possible source of variation in tracer uptake. The spatial resolution of SPECT may also be a limitation in evaluating some of the smaller regions of the brain and partial volume effects could be another source of error in the current study and the results should be interpreted with caution.

[^99m^Tc]HMPAO uptake occurred in conscious mice under controlled conditions, representing a baseline state of brain perfusion. Future studies focused on evaluating global/regional patterns of brain perfusion in 5XFAD and WT mice in response to a controlled activity paradigm prior to tracer uptake and imaging (i.e., a task that interrogates cognitive function) will be important to explore in order to further characterize disease-relevant effects on blood flow at a regional level in the brain. While SPECT studies in mice using [^99m^Tc]HMPAO have previously shown to be sensitive in detecting differences in cerebral blood flow^11^, the absence of statistically separable differences observed between 5XFAD and WT mice in the current study could relate to a basement effect in this restricted baseline state. Employing a cognitive task in the future could uncover such potential regional variations in blood flow in the 5XFAD model. While the current study investigated older mice (approximately 11 months), future work could establish age-related differences in cerebral perfusion in the 5XFAD model. Additionally, investigating cerebral perfusion in response to pharmacological manipulation or under controlled conditions of hypo/hypercapnia would add additional insight into the quantifiable detection sensitivity of the method and indeed, in the overall characterization of the 5XFAD model in general. There are distinct gender differences in human AD, and women have a higher risk of AD than men. The current study used pooled male and female data in 5XFAD and WT mice, however future work could additionally explore potential gender-related differences in cerebral blood flow within the 5XFAD model to add to the established gender-specific analyses in the 5XFAD literature^12,14^.

All the caveats notwithstanding, the current study results suggest an apparent disconnect between cerebral blood flow and glucose metabolism in the 5XFAD brain. It has been consistently observed that patterns of hypometabolism in the 5XFAD mouse brain are predominantly seen later in disease progression (up to ~ 13 months)^17^, but also at earlier timepoints^17,18^, and depends on the [^18^F]FDG metabolism metrics employed. These studies of brain metabolism ostensibly demonstrated global reductions in [^18^F]FDG uptake with contributions from various regions including the cerebral cortex, hippocampal formation, amygdala, basal ganglia, and thalamus^17,18^, whereas in the current study no statistically separable differences were observed between 5XFAD and WT mice in the same brain regions despite robust accumulation of β-amyloid pathology in 5XFAD mice. The interplay between brain perfusion, brain metabolism, and β-amyloid burden in the 5XFAD mouse model warrants further investigation.

These results reveal differences in patterns of brain perfusion between the stereotypical patterns of hypoperfusion in human AD and those observed in the 5XFAD mouse model. Additionally, an apparent disconnect has been established between cerebral blood flow and glucose metabolism in the 5XFAD mouse brain. Establishing baseline perfusion patterns in the 5XFAD model is essential to identifying differences from human AD to inform pre-clinical diagnostic and therapeutic drug discovery programs.

## Materials and methods

Formal approval to conduct the current experiments was obtained from the the Dalhousie University Committee on Laboratory Animals (Protocol No. 15-070) and the Dalhousie University Radiation Safety Committee, overseen by the Canadian Nuclear Safety Commission (License No. 07154-2-17.10). [^99m^Tc]HMPAO was synthesized at the Department of Diagnostic Imaging, Nova Scotia Heath using the precursor kit obtained as an unconditional gift from GE Healthcare Canada Inc (Mississauga, ON, Canada).

### Animals

Pairs of female WT (C57BL/6 J, Stock Number: 034848-JAX-WT-F) and male transgenic hemizygous 5XFAD mice (B6.Cg-Tg(APPSwFlLon,PSEN1*M146L*L286V)6799Vas/Mmjax, Stock Number: 034848-JAX-HET-M) were obtained from the Mutant Mouse Resource & Research Centers and cared for according to the guidelines set forth by the Canadian Council on Animal Care and as described previously^14^. The WT strain (but not WT littermates) and 5XFAD mice, obtained by continuous breeding on C57 background, were used for imaging experiments. All mice were genotyped for *APP* and *PSEN1* genes as done previously^14^.

Mice were housed in same-sex groups of 1–5, within polyethylene cages (30 × 19 × 13 cm) containing a wood-chip bedding and covered by a metal cage top and micro-isolator filter. Food (5001 Rodent Laboratory Chow, Purina, Canada) and tap water were available ad libitum. Animals were kept in a normal light/dark cycle. All experiments were carried out in accordance with Animal Research: Reporting of In Vivo Experiments (ARRIVE) guidelines and formal approval to conduct these experiments was obtained from the Dalhousie University Committee on Laboratory Animals. As done previously, imaging was performed during the light phase of the light–dark cycle^19^. A total of nine 5XFAD (M = 6, F = 3) and eight WT (M = 6, F = 2) mice with an average age of 11.5 ± 0.2 months were imaged. The number of animals studied was chosen empirically since the effect size is not known and hence power calculations could not be done. This age group was investigated to ensure there was significant neuropathology in the brains of 5XFAD mice to maximize the chances of observing potential differences in perfusion, if they exist.

### [^99m^Tc]HMPAO synthesis

Synthesis of [^99m^Tc]HMPAO followed standard procedures as described by the manufacturer (GE Healthcare Canada Inc, Mississauga, ON, Canada).

### SPECT-CT imaging

Immediately prior to imaging, mice were weighed (27.9 ± 5.5 g; 5XFAD, 31.1 ± 4.5 g; WT, 24.8 ± 4.6 g; p = 0.003). The difference in weights between the two strains was statistically significant, however, SUV and SUVR analysis (see below) are metrics normalized to injected dose per body weight, and therefore this difference had no effect on the analysis of the data. Mice were anaesthetized with 3% isofluorane in an induction chamber, and restrained in a Tailveiner Restrainer (Braintree Scientific, Braintree, MA, USA) while under a continuous stream of 1.5% isofluorane^19^. A catheter line was placed in the lateral tail vein and mice subsequently received an average dose of 44.0 ± 2.0 MBq of [^99m^Tc]HMPAO in 205-260µL saline (5XFAD, 39.4 ± 4.0 MBq; WT, 49.1 ± 8.5.0 MBq; p = 0.016). Although the injected dose was significantly different between the two strains, this difference did not have an effect on data analyses since SUV and SUVR analysis (see below) are metrics normalized to injected dose per body weight. Injection of [^99m^Tc]HMPAO was followed by a 10 μL saline flush. Mice were allowed to awaken and were returned to a separate cage, unrestrained, permitting tracer uptake to occur in conscious mice over 20 min under controlled conditions ensuring the same environmental stimulus for each animal prior to imaging. Mice then were secured in a prone position in a heated Magnetic Resonance (MR)-compatible bed, wrapped in a blanket and maintained under continuous stream of 1.5–2% isofluorane anaesthesia and the respiration rate was monitored for the duration of the scan (SA Instruments Inc., Stony Brook, NY, USA) as described previously^19^. The head region of each mouse was centered on a 14 mm axial field-of-view (FOV) and a 3-dimensional (3D) static SPECT scan was acquired in super list mode (SLM) over 45 min (4 projections) on a SPARK™ SRT-50 single head standalone tabletop SPECT scanner (Cubresa Inc., Winnipeg, MB, Canada) integrated with a Triumph XO LabPET pre-clinical CT scanner (Trifoil Imaging, Chatsworth, CA, USA)^19^.

CT scans (70kVp; 160uA beam current; 512 projections; 4 summed frames/projection; 2 × 2 binning; 2.26magnification) were obtained and collected over 8.5 min after SPECT imaging for anatomical reference and subsequently co-registered with a separate anatomical MRI scan (see below), as done previously^19^.

MRI scans were acquired in separate sessions prior to SPECT/CT imaging to enable regional analyses of brain radiotracer retention^19^. MR imaging was carried out as described previously, acquiring 142 μm isotropic images at 3.0 T over 61 min using a 3D balanced Steady-State Free Precession, (b-SSFP) imaging sequence (T2/T1-weighting)^19^.

### Image processing

SPECT images were reconstructed as previously done, with SPECT SLM data converted to list mode data using a built-in Cubresa SPARK™ preprocessing routine at 140 keV with a 20% energy window applied^19^. The list mode data were reconstructed using an iterative 3D Maximum-Likelihood Expectation Maximization (MLEM) algorithm (9 iterations) using HiSPECT software (SciVis GmbH, Göttingen, Germany)^19^. The resulting SPECT images produced an effective in-plane resolution of 0.8 mm. As described previously, dark image and quantitative calibrations were performed weekly for the duration of the study and applied to each image acquired^19^.

Reconstruction of CT images was carried out as previously described with a 512 × 512 × 512 image matrix over a 56 mm FOV using built-in optimum noise reconstruction procedures with the Triumph XO CT acquisition software, providing images with 102 μm isotropic resolution^19^. SPECT and CT images were fused using established coordinate transformations between two modalities, whose common coordinate frames were applied in AMIDE Imaging Analysis software^19,20^. All images were visually inspected to ensure fusion results were correct^19^. As noted previously, MRI images underwent 3D maximum intensity projection (MIP) processing of 4 phase cycle frequencies and the resulting reconstructed images were zero-padded, interpolated to a higher resolution grid to increase the effective resolution and image quality, in ImageJ (National Institutes of Health, Bethesda, MD, USA)^19^.

### SPECT/CT/MRI coregistration and SPECT regional analysis

SPECT/CT/MRI scans underwent inter-modality registration and the brain was parcellated using a MR-based 3D digital mouse atlas for regional analyses as described previously^17,19^. A 6-parameter rigid body registration was carried out using an established method^19^ between the mouse MR and a standard brain from which the digital atlas was derived using Automated Image Registration 5.3.0^21^. Higher spatial transformations (warping) were applied to the standard brain and corresponding warped MR atlas^19^. All MRI, warped MR atlas, and SPECT/CT-fused images were imported into AMIDE to affine registration between modalities^19,20^.

To evaluate brain perfusion, SPECT volume of interest (VOI) statistics were generated for six brain ROIs defined by the MR atlas: (i) whole brain (excluding cerebellum, brain stem, and olfactory bulb), (ii) neocortex, (iii) hippocampal formation, (iv) amygdala, (v) thalamus, and (vi) basal ganglia. Semiquantitative estimates of perfusion were determined for each of the ROIs via SUV, expressed as SUV = (activity/unit dose)/g. SUVR were also evaluated, using a whole brain (wb) reference region, determined as follows: SUVR_roi_ = SUV_roi_/SUVR_wb_. This normalized metric was employed to reduce any possible inter-subject or inter-scan variability in the study^19^.

### β-amyloid immunohistochemistry

After imaging, 5XFAD and WT brains tissues were processed and stained for β-amyloid using standard immunohistochemical techniques as previously described^14^ to detect β-amyloid deposition. Staining was done using a primary antibody for polyclonal rabbit anti-β-amyloid (1:400; Cat No. 71-5800, Invitrogen, Rockford, IL, United States)^14^. Stained mouse brain sections were analysed and photographed using a Zeiss Axio Scan.Z1 slide scanner with Zen 3.1 Blue Edition software (Carl Zeiss Canada Ltd, Toronto, Canada). Using Adobe Photoshop (CS 5, Version 12.0, San Diego, CA, United States), photomicrographs were assembled.

### Statistical analysis

Average SUV and SUVR brain perfusion metrics were compared between 5XFAD and WT group means for each brain ROI using unpaired t-tests (two tailed, assuming unequal variances). Differences were concluded at a significance level of 5% (p < 0.05,*)^19^. All data are presented as group means ± standard deviation (SD). Statistical tests were performed in SPSS (IBM, Armonk, NY, USA).

## Data Availability

Full anonymized data will be shared by the corresponding author (S.D.) at the request from any qualified investigator.
